# The RNA Helicase Rm62 Cooperates with SU(VAR)3-9 to Re-Silence Active Transcription in *Drosophila melanogaster*

**DOI:** 10.1371/journal.pone.0020761

**Published:** 2011-06-02

**Authors:** Joern Boeke, Indira Bag, M. Janaki Ramaiah, Irene Vetter, Elisabeth Kremmer, Manika Pal-Bhadra, Utpal Bhadra, Axel Imhof

**Affiliations:** 1 Munich Center of Integrated Protein Science and Adolf-Butenandt Institute, Ludwig Maximilians University of Munich, Munich, Germany; 2 Centre for Chemical Biology, Indian Institute of Chemical Technology, Hyderabad, India; 3 Functional Genomics and Gene Silencing Group, Centre for Cellular and Molecular Biology, Hyderabad, India; 4 Institute of Molecular Immunology, Helmholtz Zentrum Munich, Munich, Germany; Institute of Genetics and Molecular and Cellular Biology, France

## Abstract

Gene expression is highly dynamic and many genes show a wide range in expression over several orders of magnitude. This regulation is often mediated by sequence specific transcription factors. In addition, the tight packaging of DNA into chromatin can provide an additional layer of control resulting in a dynamic range of gene expression covering several orders of magnitude. During transcriptional activation, chromatin barriers have to be eliminated to allow an efficient progression of the RNA polymerase. This repressive chromatin structure has to be re-established quickly after it has been activated in order to tightly regulate gene activity. We show that the DExD/H box containing RNA helicase Rm62 is targeted to a site of rapid induction of transcription where it is responsible for an increased degree of methylation at H3K9 at the heat shock locus after removal of the heat shock stimulus. The RNA helicase interacts with the well-characterized histone methyltransferase SU(VAR)3-9 via its N-terminus, which provides a potential mechanism for the targeting of H3K9 methylation to highly regulated genes. The recruitment of SU(VAR)3-9 through interaction with a RNA helicase to a site of active transcription might be a general mechanism that allows an efficient silencing of highly regulated genes thereby enabling a cell to fine tune its gene activity over a wide range.

## Introduction

Gene expression is regulated at the level of initiation, elongation and termination of transcription [1], [2]. In order to modulate the expression of a given gene, basal as well as sequence specific transcription factors cooperate to facilitate the recruitment of the RNA polymerase to a given promoter and regulate its activity. Besides the mere DNA sequence of the regulatory regions, the wrapping of DNA into chromatin heavily influences the level to which a particular gene is transcribed [3], [4]. The degree of chromatin packaging can be modulated by histone modifying enzymes that generate a specific modification pattern thereby marking active and inactive genes [5], [6]. Although the modification patterns can allow a distinction between genes that are permanently silenced and those that are actively transcribed, it is unclear how genes that can cycle between a highly active and an inactive state are marked. Such genes often respond to an external signal such as a hormone or intracellular stress [7]. The signal is usually perceived by a specific transcription factor that associates with a promoter via binding to a specific DNA element in the regulatory region of the gene. Along with the transcription factor, multiple transcriptional co-activators are recruited that can either facilitate the binding of the basal transcriptional machinery, modify histones [8], remodel nucleosomes or displace nucleosomes from the whole genomic locus [9]. When the signal ceases, nucleosomes reform at the locus and the repressed state is reconstituted. Although there is some controversy of whether or not the formation of a nucleosomal structure is a cause or a consequence of repression [10] there is a clear correlation between the two events [11].

One of the best-characterized promoters that can rapidly switch between an active and an inactive state controls the transcription of the *hsp70* gene. The promoter of the *hsp70* gene adopts a well defined chromatin structure that is hypersensitive towards DNaseI [12], [13], [14] and has TBP bound [15]. This specific promoter architecture leads to a recruitment of RNA polymerase II even in absence of the stimulus and the generation of a paused polymerase that requires a stimulus to be released from the pre-initiation complex [16], [17], [18], [19]. A heat shock pulse then leads to a cooperative binding of the sequence specific transcription factor HSF1 [20], [21], [22] to the *hsp70* promoter, which in turn results in promoter clearance of RNA polymerase II and the subsequent accumulation of *hsp70* RNA. The heat shock pulse also leads to a recruitment of histone modifying enzymes such as the H3K4 methyltransferase PAF1, which results in an increase of H3 methylated at K4 over the whole body of the gene [8] and histone chaperones such as FACT that facilitate nucleosome disassembly [23], [24]. As a consequence the nucleosomes get disrupted over a large region of the *hsp70* locus [9] allowing transcription to occur at a high level. This process of histone removal is dependent on the poly ADP ribosyltransferase (PARP) and is independent of processive transcription [9], [25].

Despite this large body of knowledge with regards to *hsp70* activation, little is known about the mechanisms that re-establish the repressed state at this highly inducible gene. Transcriptionally inactive genes are frequently marked by a methylation of H3 at position K9. This modification is catalyzed by H3K9 specific methyltransferases such as SU(VAR)3-9 [26], G9a [27] or SETDB1 [28]. Although this modification localizes predominantly to pericentric heterochromatin, H3K9 methylation has also been detected at euchromatic regions [29], [30] and has been suggested to be important for transcriptional activation [31], [32]. However, the activating function of H3K9me seems to be an exception from the rule as targeted methylation of H3K9 to ectopic sites lead to the generation of silenced chromatin [33]. Currently one of the best-characterized histone methyltranseferases is SU(VAR)3-9 [26]. It has been initially identified by a genetic screen for factors that affect heterochromatin formation in *Drosophila* [34], [35] and has been subsequently identified in many higher eukaryotes [36]. All SU(VAR)3-9 orthologoues have a conserved domain structure containing a chromo domain at the N-terminus and a catalytically active C-terminal SET domain. Besides the catalytically active SET domain, the region that resides N-terminal of the chromo-domain of SU(VAR)3-9 also plays an important role in modulating SU(VAR)3-9s methyltransferase activity [37], [38]. We therefore analyzed proteins that interact with the SU(VAR)3-9 N-terminus in order to find potential regulators of SU(VAR)3-9 and understand the mechanism of how SU(VAR)3-9 exerts its function in eu- as well as in heterochromatin. One of the proteins we have identified as interactor with the N-terminal tail of SU(VAR)3-9 is the DExD/H containing RNA-helicase Rm62. Rm62 plays a role in multiple gene regulatory processes such as alternative splicing RNA release and subsequent export [39], steroid receptor mediated activation of transcription [40], [41] and RNAi mediated silencing [42], [43]. We show that Rm62 interacts with SU(VAR)3-9 N-terminal region *in vitro* as well as *in vivo*. As Rm62 has been reported as a mediator of *hsp70* transcriptional shut down, we also investigated the function of SU(VAR)3-9 in this process and find a similar role like Rm62. We observe an increase in H3K9 methylation during transcriptional shut down on the heat shock loci on polytene chromosomes that is dependent on the presence of Rm62 suggesting a functional role of the RNA helicase on recruiting a methyltransferase.

## Materials and Methods

### Affinity purifiction of Proteins binding to the SU(VAR)3-9 N-terminus

GST and GST SU(VAR)3-9 NT (aa 1–152) were expressed in *E. coli* and individually bound to GSTrap FF columns (GE Healthcare). The columns A (GST) and B (GST SU(VAR)3-9 NT) were connected and a *Drosophila* nuclear extract from 0–12 hours embryos (NE) was loaded. After a washing step (200 mM NaCl, 20 mM Tris-HCl, (pH 8.0), 1 mM EDTA, 0.5% Nonidet P-40), the columns A and B were disconnected followed by step elution (250, 500 and 750 mM) of the bound proteins on a ÄKTA-FPLC system (GE Healthcare). Fractions were analyzed for bound proteins by fractionation on SDS-polyacrylamide gel electrophoresis followed by silver or Coomassie staining. Stained protein bands were cut out and subjected to mass spectrometry.

### Generation of Rm62 specific rat monoclonal antibodies

Rm62 was expressed in *E.coli* as an N-terminal fusion protein with the Glutathion-S-Tranferase (GST) (cloning details are available on request). Immunization was performed in the “Service Unit Monoclonal antibodies” at the Helmholtz Zentrum München, using purified GST-Rm62. A ELISA screen led to thirthy-two (GST negative, Rm62 positive) hybridoma supernatants, which were re-screened for their specificity in western blots and immunoprecipitations. The two antibodies used in the present study were either of IgG1 (1B8) or of IgG2c (1E7) subtype.

### GST pull-down of in vitro translated proteins

GST and GST fusion proteins were expressed in *E. coli*. GST pull-downs were carried out essentially as described earlier [44]. Bacteria were induced with 0.2 mM isopropyl-D-thiogalactopyranoside (IPTG) for 3 h at 37°C. Recombinant proteins were purified with glutathione-sepharose beads (GE Healthcare) and analyzed by SDS-PAGE to normalize protein amounts. Equivalent amounts of GST fusion proteins were incubated with [^35^S]-methionine-labeled proteins, produced by the T7/T3 TNT-coupled transcription/translation system (Promega) in 200 µl of binding buffer (100 mM NaCl, 20 mM Tris-HCl (pH 8.0), 1 mM EDTA, 0.5% Nonidet P-40, 5 µg of ethidium bromide, 100 µg of bovine serum albumin (BSA)). After 0.5 h of incubation at room temperature, the beads were washed 5 times with 1 ml of binding buffer without ethidium bromide and BSA. The bound proteins were eluted with SDS sample buffer, separated by SDS-PAGE, and visualized by autoradiography.

### Cell culture and Immunoprecipitation

*Drosophila* Schneider cells (SL2), stably transfected with an expression plasmid coding for a haemaglutinin-(HA-) tagged version of SU(VAR)3-9 under control of a metallothionein promoter (cloning details are available on request), were grown in Schneider's Drosophila medium (Gibco) +10% fetal calf serum at 26°C. 12 hours before harvesting expression of HA-SU(VAR)3-9 was induced by the addition of 0.2 mM CuSO_4_ to the media. Nuclear extracts (NE) of these cells were subjected to immunoprecipitations using the a-Rm62 rat monoclonal antibody 1E7 (monoclonal rat antibody, IgG2c subtype): equal amounts of NE weres incubated for three hours with the 1E7 antibody or with an unspecific antibody of the same isotype (IgG2c), respectively. For immunoprecipitation 20 µl of a 1∶1 mixture of Protein A/G Sepharose (GE Healthcare) was added to the extracts and incubated for 3 hours at 4°C. After washing the Protein A/G beads with BC-300 (300 mM NaCl, 25 mM HEPES pH 7.6, 1 mM MgCl_2_, 0.5 mM EDTA, 0.5 mM EGTA, 10% Glycerol) including 0.1% NP-40, bound proteins were eluted by the addition of SDS sample buffer and subjected to SDS-gelelectrophoresis followed by western blot using HA-tag (Roche), SU(VAR)3-9 (Su3D9, IgG1 subtype) and Rm62 specific antibodies (1B8, monoclonal rat antibody, IgG1 subtype).

### Reverse Transcription (RT) and Realtime PCR

Total cellular RNA from SL2 cells of was isolated using the RNeasy Mini Kit (Qiagen). Isolated RNA was cleaned up by DNase treatment with the RNase-Free DNase Set (Qiagen) to avoid possible DNA contaminants. 100 ng of RNA were taken for the first strand cDNA synthesis using M-MuLV Reverse Transcriptase (New England Biolabs) and gene specific primers for *hsp70* and U6 snRNA (internal control). Q-PCR was carried out using the ABI PRISM 7000 Sequence detection system (Applied Biosystems). SYBR Green 2× PCR Master Mix (Applied Biosystems) was used according to the manufacturer's directions.

To control the efficiency of the knockdown, total cellular RNA from the RNAi treated SL2 cells were isolated using the RNeasy PLUS Mini Kit (Qiagen), 6 days after RNAi treatment. 1 µg of total RNA were taken for the first strand cDNA synthesis using M-MuLV Reverse Transcriptase (New England Biolabs) and gene specific primers for *Su(var)3-9* and *Rm62*, respectively. 10% of the RT reaction was used for standard PCR with exon specific primer pairs (Su(var)3-9RT_for: 5′-CGGTCATGTGGCTCACGGCAA-3′, Su(var)3-9RT_rev: 5′-GGCGGCGGAATCGGCTAT GT-3′; Rm62RT_for: 5′-GTGCTGGACGAGGCCGATCG-3′, Rm62RT_rev: 5′-GCGGATGA AGCGCACCAGGT-3′) followed by agarose gel electrophoresis. The knock downs had no effect on cell division and growth (Figure S1).

For the analysis of *hsp70* RNA in flies, total cellular RNA from larvae of different *Drosophila* stocks was isolated using Trizol (Invitrogen). RNA was purified by a RNeasy Mini Kit (Qiagen). Three µg of RNA were used for the first strand cDNA synthesis using SuperScript™ first-strand synthesis system for RT-PCR (Invitrogen). The cDNA was further used for the quantification of gene expression by Real-Time PCR by ABI 7500 Instrument.

### Chromatin-Immunoprecipitation (ChIP) from *Drosophila* larvae

*Drosophila* wildtype and *Rm62* mutant (*CBO2119/LIP-F*) larvae were grown in standard food media. Approximately 200–300 mg of well-fed third instar larvae was used for each reaction. Larvae were heat shock treated at 37°C for 45 min and were sacrificed immediately. For the recovery after heat shock, larvae were further cultured in normal temp (25°C) for another three hours. All larvae were resuspended in 5 ml of buffer A1 (60 mM KCl, 15 mM NaCl, 4 mM MgCl2, 15 mM HEPES (pH7.6), 0.5% Triton X-100, 0.5 mM DTT, 10 mM sodium butyrate, EDTA-free complete protease inhibitor cocktail (Roche) and crosslinked with 1.8% formaldehyde at room temperature. To stop the cross linking reaction, 2.5 M glycine was added to a final concentration of 225 mM, mixed thoroughly and further incubated for 5 min on ice. The cross-linked larvae ware resuspended in 2.5 ml of lysis buffer (140 mM NaCl, 15 mM HEPES pH 7.6, 1 mM EDTA, 0.5 mM EGTA, 1% Triton X-100, 0.5 mM DTT, 0.1% sodium deoxycholate, 0.05% SDS, 10 mM sodium butyrate, EDTA-free complete protease inhibitor cocktail (Roche) 0.1% SDS and 0.5% N-lauroylsarcosine and incubated for 10 min at 4°C. Chromatin was sheared in a Bioruptor (Diagenode) to achieve chromatin fragments of an average length from 300–500 bp. The sonicated chromatin was diluted with ChIP dilution buffer (0.01% SDS, 1.1% Triton-X100, 1.2 mM EDTA, 16.7 mM Tris-HCl pH 8.1, 167 mM NaCl, 1× EDTA free protease inhibitors cocktail (Roche)) and pre-cleared with Protein A agarose/salmon sperm DNA beads (Millipore) for 1 hour at 4°C. About 200 µg of precleared chromatin was incubated with 25 µl of anti-Rm62 (1E7), anti-H3K9me2 (Upstate 07-212) and anti-SU(VAR)3-9 (SU3D9) overnight prior to the addition of a protein A Sepharose beads. Immunoprecipitated complexes were washed sequentially with low salt buffer (0.1% SDS, 1% Triton X100, 2 mM EDTA, 20 mM Tris-HCl pH8.1, 150 mM NaCl), high salt buffer (0.1% SDS, 1% Triton –X100, 2 mM EDTA, 20 mM Tris-HCl pH8.1, 500 mM NaCl), LiCl wash buffer (0.25 M LiCl, 1% NP-40, 1% Desoxycholate, 1 mM EDTA, 10 mM Tris-HCl pH8.1) and 10 mM Tris-HCl, 1 mM EDTA pH8 (2 times). The bound DNA was eluted with elution buffer (1% SDS, 0.1 M Na_2_HCO_3_) and cross links removed for 6 hrs at 65°C. After treatment with RNase A (Sigma) and Proteinase K (Sigma), DNA was purified with Nucleospin Extract II DNA purification columns according to manufacturer's instructions (Macharey Nagel). The sample was amplified following a standard PCR protocol using primers covering the *hsp70* promoter (forward: 5′-TGCCAGAAAGAAAACTCGAGAAA, reverse: 5′-GACAGAGTGAGAGAG CAATAGTACAGAGA). The ratios of amplified immunoprecipitated DNA and DNA amplified from 5% of the input material were calculated from triplicate gels by densitometry.

### Immunostaining of polytene chromosomes

Salivary glands of third Instar larvae were dissected and fixed in 4% Para formaldehyde. The polytene chromosomes were further processed and immunostained with antibodies as described earlier [45]. Chromosomes were probed with anti Rm62 antibodies at a dilution 1∶30, Cy3-conjugated goat anti rat secondary antibodies were used for Rm62 at a standard 1∶200 dilution. For H3K9me2 staining, chromosomes were immunostained with anti-H3K9me2 antibodies (1∶25) and re-probed with Cy5 conjugated goat anti-rabbit antibodies at a 1: 200 dilution. The chromosomes were mounted with vecta-shield mounting media with Propidium Iodide and examined in Olympus FV1000 confocal microscope using a ×60 water immersion lens.

## Results

### SU(VAR)3-9 interacts with a component of the RNAi machinery

**Figure 1:**
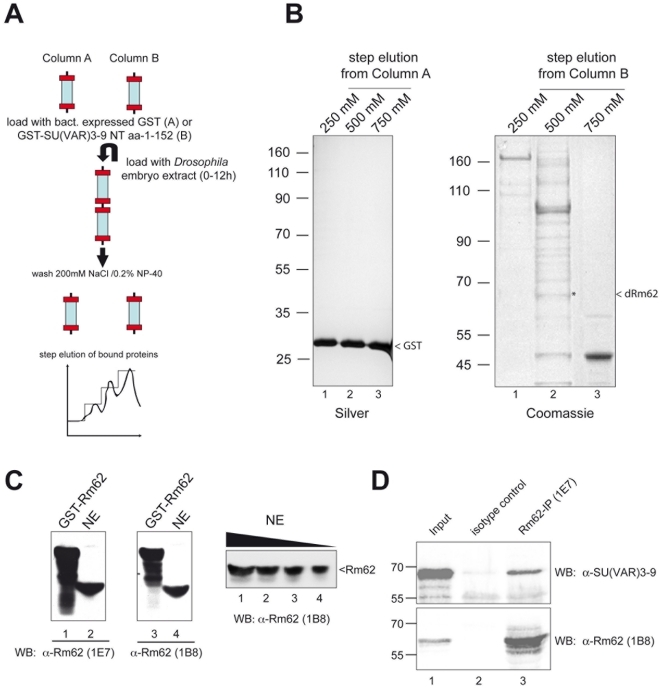
Identification of the DEAD-box RNA helicase Rm62 as an interaction partner of SU(VAR)3-9. (**A**) Schematic representation of the purification scheme used (**B**) Rm62 binds to the SU(VAR)3-9 N-terminus. *left panel:* Silverstain of a SDS-polyacrylamide gel loaded with salt eluted fractions from column A *right panel:* Coomassie staining SDS-polyacrylamide gel loaded with salt eluted fractions from column B. The band marked with the asterisk (*) was identified as *Rm62* via mass-spectrometry. (**C**) The rat monoclonal antibodies 1B8 and 1E7 are specific for Rm62. *Left:* Western Blot analysis of recombinant and endogenous Rm62 probed with 1B8 or 1E7. Lane 1: purified recombinant GST-Rm62. Lane 2: 5 µl of a nuclear extract (NE) from *Drosophila* embryos (0–12 h a.e.l.). *Right:* Decreasing amounts of NE (lane 1–4: 10 µl, 5 µl, 2.5 µl, 1 µl) were separated by SDS-PAGE and subjected to Western Blot analysis using 1B8. (**D**) SU(VAR)3-9 copurifies with Rm62 in immunoprecipitations using the Rm62 specific antibody 1E7. NE of copper induced (0.2 mM) HA-SU(VAR)3-9 expressing cells were used for co-immunoprcipitation (Co-IP) experiments. NEs were incubated with 1E7 antibody and Rm62 was purified by the addition of Protein A/G sepharose beads (GE Healthcare). Bound proteins were eluted with SDS sample buffer and subjected to gel electrophoresis followed by western blotting (Input: 5% of the amount of NE used for the Co-IP, beads: unspecific control using either an antibody of the same isotype (isotype control, IgG2c) Rm62-IP (1E7): Co-IP with the 1E7.

**Figure 2:**
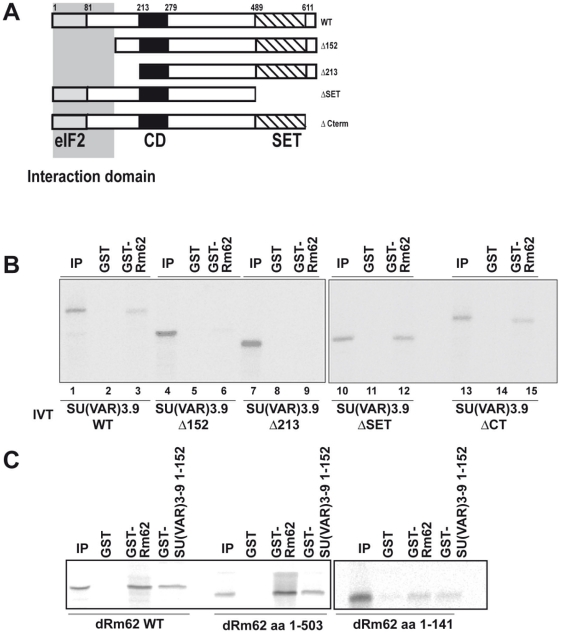
Mapping of the interaction domain between SU(VAR)3-9 and Rm62. (**A**) Schematic display of the *in vitro* translated SU(VAR)3-9 proteins. (**B**) The various *in vitro* translated SU(VAR)3-9 constructs were incubated with GST (GST) or a GST-Rm62 fusion protein to map the interaction domain. (**C**) In a reciprocal experiment GST-Rm62 and a GST-SU(VAR)3-9 containing only the 152 N-terminal amino acids were used as baits for *in vitro* translated Rm62.

As the N-terminus of SU(VAR)3-9 plays an important functional role [35], [37], [38], [46], we expressed the N-terminal domain of SU(VAR)3-9 as a GST fusion protein (Figure 1A) and used it as an affinity resin to purify interacting partners of SU(VAR)3-9. Among other proteins [47], we have identified the DExD/H box containing RNA helicase Rm62 as a specific interactor with SU(VAR)3-9 (Figure 1B). In order to confirm the interaction *in vivo* we generated specific monoclonal antibodies recognizing Rm62 (Figure 1C) and used it to immunoprecipitate a putative Rm62 complex. As the concentration of endogenous SU(VAR)3-9 are so low that the protein is difficult to detect, we performed the immunoprecipitation in a cell line that expressed HA-tagged SU(VAR)3-9 under a copper inducible promoter. In this experiment we could detect SU(VAR)3-9 co-immunoprecipitating with Rm62 using a Rm62 specific antibody but not with a control antibody (Figure 1D). As the observed interaction could have been mediated by intermediary factors we checked if we could observe the interaction *in vitro* by mixing GST fusion proteins of Rm62 and SU(VAR)3-9 (Figure 2) with various deletion mutants of Rm62 or SU(VAR)3-9 translated *in vitro*. As expected from the pull down experiments shown in Figure 1B, N-terminal truncations of SU(VAR)3-9 abolished its interaction with immobilized recombinant GST-Rm62 protein whereas C-terminal truncations can still interact (Figure 2B). This direct interaction is mediated by the first 141 amino acids of Rm62, which do not contain the helicase domain (Figure 2C). The same domain is also responsible for the dimerisation of Rm62 suggesting that it may be an important region for regulating this protein-protein interaction. However, a more detailed analysis would be required to determine the molecular function of this region of Rm62.

### SU(VAR)3-9 and Rm62 regulate *hsp70* transcriptional shutdown

**Figure 3:**
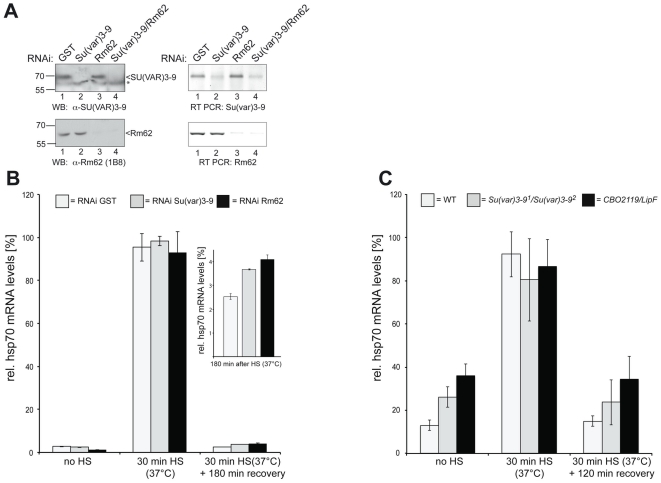
Prolonged RNA production from the *hsp70* locus after reduction of SU(VAR)3-9 or Rm62. (**A**) RNAi against SU(VAR)3-9 and/or Rm62 specifically eliminates the respective proteins as well as it's mRNA from SL2 whole cell extract (WCE). SL2 cells were transfected with specific double stranded RNAs against SU(VAR)3-9, Rm62 or GST (control). WCEs and total RNA were prepared 6 days after transfection. *Left*: Proteins were analyzed by SDS-PAGE followed by western blotting with the indicated antibodies (GST: control RNAi, SU(VAR)3-9: RNAi against Su(var)3-9, Rm62: RNAi against Rm62, SU(VAR)3-9/Rm62: double knockdown of Su(var)3-9 and Rm62). *Right*: 1 µg oft total RNA, was reverse transcribed (Superscript™, Reverse Transcriptase, Invitrogen) using gene specific primers for Su(var)3-9 or Rm62, respectively. 10% of the obtained cDNA were analyzed by standard PCR using specific primers for Su(var)3-9 or Rm62, and separated by agarose gel electrophoresis. To discriminate between genomic and cDNA, we used intron spanning primer pairs in the PCR reaction. (**B**) Quantitative RT-PCR of the *hsp70 m*RNA from SL2 cells before (no HS), after heat shock (30 min HS) and a 180 min of recovery phase (+180 min recovery). SL2 cells, cultured under standard conditions, were subjected to RNAi against GST (control), Su(var)3-9 or Rm62. After 6 days of culturing, cells were either not treated (no HS) or treated with a heat shock (30 min HS) followed by a recovery for 180 min at 26°C (+180 min recovery), respectively. RNA from these cells was isolated, reverse transcribed and analyzed by quantitative real time PCR. Bars represent relative *hsp70* RNA expression levels (in percent) normalized to an internal control (U6 snRNA), which does not respond to heat shock. Percent expression was calculated to the maximal amount of RNA measured after heat shock. The inlet graph shows an enlargement of the calculated values after 180 min recovery upon heat shock. The observed difference between the SU(VAR)3-9/GST and Rm62/GST is significant as calculated with an unpaired two sided Student's t-test (p = 0.041 and p = 0.014, respectively). Error bars indicate the standard deviation of two replicates. (**C**) Relative expression of *hsp70* RNA from wild type (WT) Rm62 (*CBO2119/LipF*) and Su(var)3-9 heteroalleic flies (*Su(var)3-9^1^/Su(var)3-9^2^*) before (no HS) or after heat shock (30 min HS) followed by 120 min of recovery (+120 min recovery). RNAs were extracted from the flies either not treated or treated with a heat impulse and “recovered” for 120 min at 25°C and further subjected to quantitative real time PCR. Bars represent relative *hsp70* RNA expression levels (in percent) normalized to an internal control (18S rRNA), which has a minimal effect on heat shock. The observed difference between Su(var)3-9 mutant and Rm62 mutants compared to wildtype flies is significant as calculated with an unpaired two sided Student's t-test (p = 0.047 and p = 0.003, respectively). Error bars indicate the standard deviation of three replicates.

As we see an interaction between Rm62 and SU(VAR)3-9 we wondered whether they may also regulate similar genes. Rm62 has been shown to be involved is the shut down of the *hsp70* gene after heat shock. Therefore, we subjected SL2 cells to a 30 minute heat shock at 37°C and determined the relative levels of hsp70 RNA after recovery from heat shock by quantitative RT-PCR (Figure 3B). In accordance to what has been shown before [39] we see a significant delay in the transcriptional shut down of *hsp70* transcription in cells where Rm62 has been removed by RNAi (Figures 3A, bottom panel and B). When we knock down Su(var)3-9 transcription (Figure 3A, top panel), we observe a similar effect (Figure 3B) suggesting that SU(VAR)3-9 also has a role in re-silencing the *hsp70* gene. The increased *hsp70* transcription is not due to an increased heat shock response, as we do not detect significant changes in the amount of *hsp70* RNA immediately after heat shock (Figure 3B). We next wondered whether we would see the same effect of Rm62 and SU(VAR)3-9 on hsp70 transcription in Drosophila larvae. To do this, we analyzed the recovery of hsp70 transcription from heat shock in flies that carry a mutation in *Rm62* or *Su(var)3-9* (Figure 3C). Similar to what we see in SL2 cells, we also observe an increased *hsp70* transcription after 3 hrs of recovery in the mutant flies when compared to wildtype. In the mutant flies we find an increased *hsp70* expression even before heat shock, suggesting that the prolonged lack of SU(VAR)3-9 or Rm62 leads to a relaxed chromatin structure of the normally tightly regulated *hsp70* locus in the corresponding mutant fly strains (Figure 3C).

**Figure 4:**
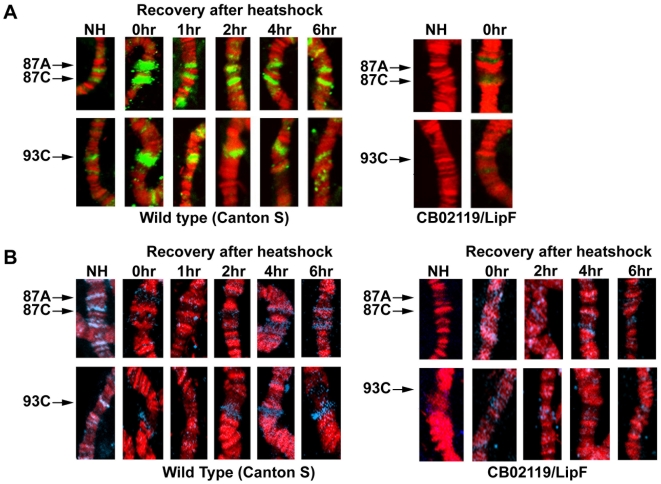
Recruitment of Rm62 and histone H3K9me2 proteins on polytene chromosomes and heat shock puffs. (**A**) The larvae were incubated at 37°C for 45 min for immediate heat shock (0 hr recovery time) and subsequently transferred to normal culture temperature (25°C). Larvae were dissected at different time points (1 to 6 hr recovery time). Non heat shocked (NH) larvae were used as controls. Immunostaining of a part of chromosome 3 covering 3 heat shock puffs (87A, 87C and 93C, using the nomenclature of [56]) with Rm62 Abs (1B8) (**A**) or H3K9me2 antibodies (blue) (**B**) from the wild type Canton S larvae before and after heat treatment (37°C at 45 min) showing endogenous H3K9me2 bindings. The difference in accumulation of H3K9me2 at the heat shock puffs was visualized by the protein signals in blue (arrow head).

**Figure 5:**
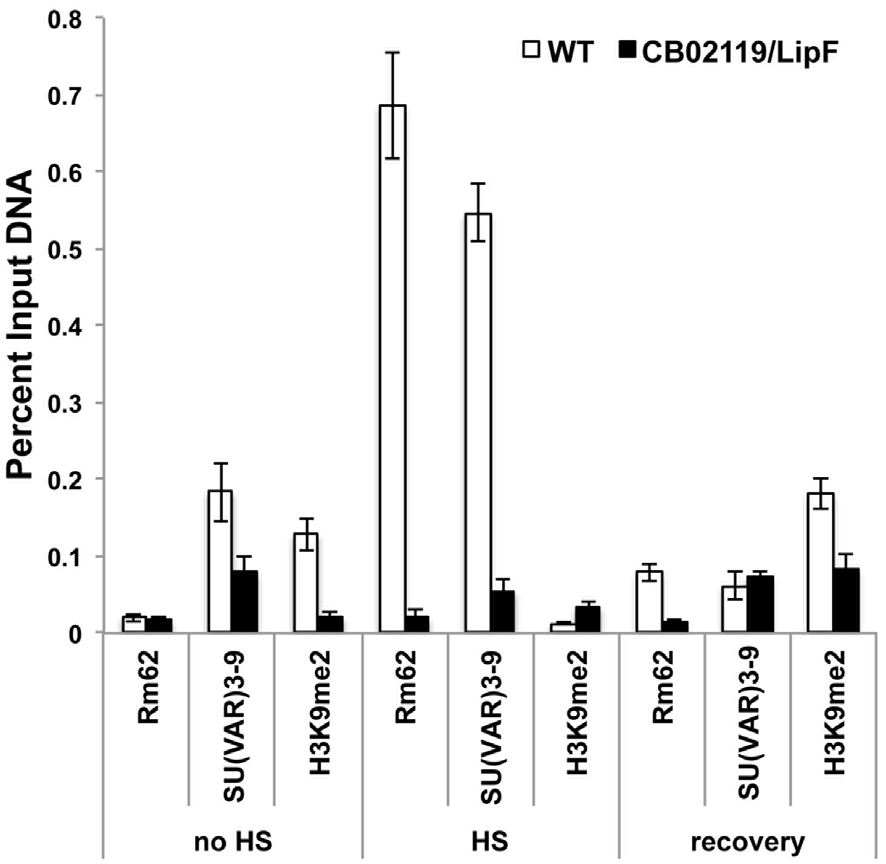
Binding of Rm62, SU(VAR)3-9 and H3K9me2 before, during and after heat shock. Chromatin immunoprecipitation comparing the binding of Rm62, SU(VAR)3-9 and H3me2K9 on the *hsp70* promoter was assayed in third instar larvae of wild type (white bars) and heteroallelic *Rm62* (*CBO2119/Lip F*) mutant strains (dark bars) either without heat shock (no HS), heat incubation of larvae at 37°C for 45 min (HS) and 3 hrs after heat treatment (recovery) . The enrichment of proteins for each amplicon of the *hsp70* locus was measured relative to the input material. The relative ratios from three independent experiments were depicted as an individual bar. For quantitation, the bands were analyzed using the Odyssey Imaging System (LI-COR Biosciences) system and expressed of percent of input DNA. Error bars indicate the standard deviation of three biological replicates.

We next wanted to know if Rm62 is indeed recruited to the *hsp70* gene *in vivo* and if this recruitment is required for the methylation of H3K9 following re-silencing. To do this, we incubated third instar larvae of wildtype or mutant flies for 45 minutes at 37°C. This heat shock resulted in puffing of the three heat shock loci 87A, 87C and 93C. Concomitantly with the heat shock Rm62 is recruited to the heat shock loci and slowly dissociates from the locus during recovery (Figure 4A). Consistent with the hypothesis that Rm62 recruits a H3K9 methyltransferase we observe a re-establishment of the H3K9me2 signal when *hsp70* transcription ceases (Figure 4B). The kinetics of H3K9me2 re-appearance are much slower than the recruitment of Rm62, which could either reflect the dynamics of histone molecules after heat shock or the presence of a demethylase immediately after heat shock. In flies that carry a null mutation of Rm62 (lipF) and fail to fully shut down *hsp70* transcription [39] (Figure 3C), the histones that reassemble on the heat shock loci have a lower degree of H3K9 methylation suggesting that Rm62 is indeed responsible for the targeting of a H3K9 specific methyltransferase (Figure 4B, right panel). In order to get a better picture of the recruitment of Rm62 and SU(VAR)3-9 to the *hsp*70 locus, we performed chromatin immunoprecipitations of the *hsp70* promoter region using antibodies against Rm62, SU(VAR)3-9 and H3K9me2 in wildtype or Rm62 mutant flies (Figure 5). Comparable to what we observe in polytene chromosomes, Rm62 is recruited to the *hsp70* promoter immediately after heat shock and is no longer crosslinked to the promoter after a 3 hr recovery phase. Likewise, SU(VAR)3-9's binding to the promoter is substantially increased after heat shock. The recruitment of SU(VAR)3-9 is dependent on the presence of Rm62 as the binding is decreased in the Rm62 mutant flies (Figure 5, dark bars). In the absence of any heat shock and after the promoter recovered from heat shock the histones carry a methylation at H3K9, which is dependent on the presence of Rm62 (Figure 5). Interestingly, despite a clear enrichment of the methylating enzyme, H3K9me2 is reduced at the *hsp70* promoter immediately after heat shock wildtype flies, which is likely due to the complete removal of histones after heat shock [9].

**Figure 6:**
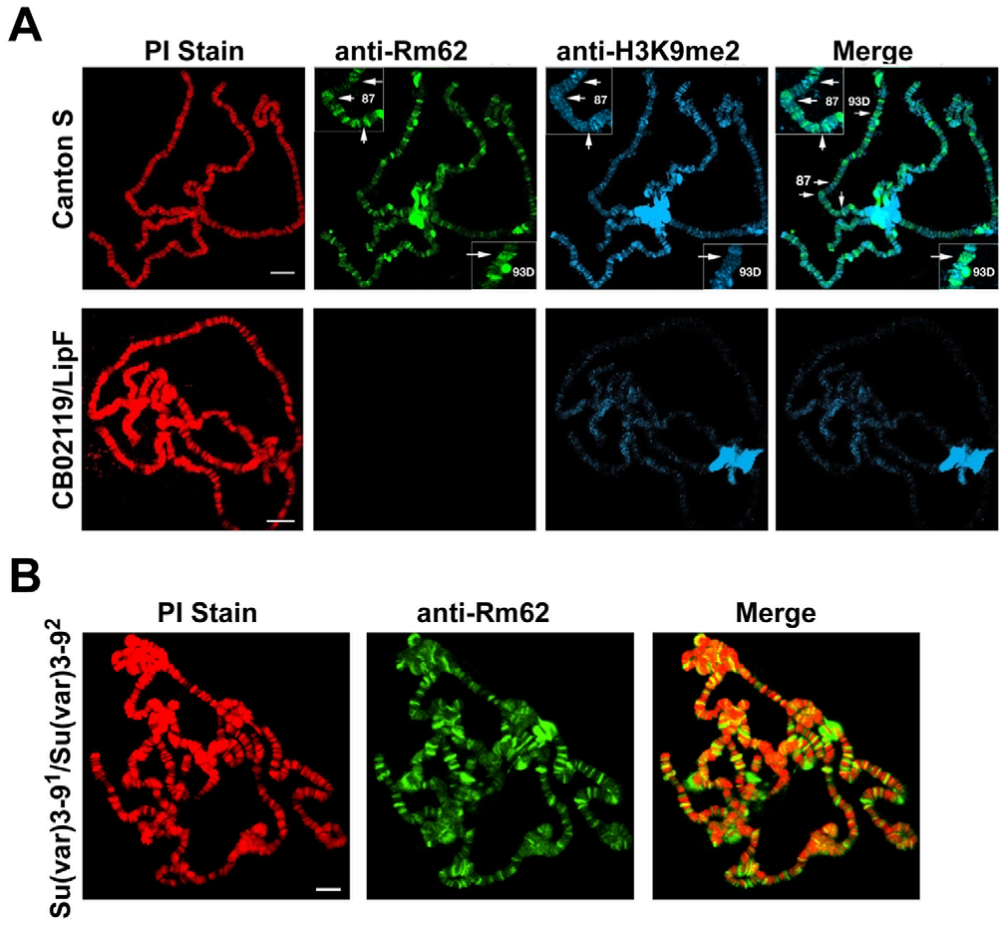
Global effect of Rm62 on H3K9me2. (**A**) Immunostaining of polytene chromosomes of third instar wildtype and heteroalleleic Rm62 (*CBO2119/LipF*) mutant larvae with Rm62 (green) and H3K9me2 antibodies (blue). The left panel shows a merge of the signals of an anti Rm62 and an anti H3K9me2 staining showing a large portion of costaining. Inserts show an enlargement of the heat shock loci on chromosome 3. The chromosomes were counterstained with PI (red) Scale −10 µm. (**B**) Immunostaining of polytene chromosomes of third instar Su(var)3-9 mutant larvae (*Su(var)3-9^1^/Su(var)3-9^2^*) with Rm62 antibodies (green) show no disturbance of Rm62 binding. The chromosomes were counterstained with PI (red) Scale −10 µm.

The recruitment of histone methylation by Rm62 is not restricted to the heat shock loci as we observe a reduction of H3K9me2 levels at many euchromatic binding sites in heteroallelic larvae carrying two mutant *Rm62* alleles (Figure 6A). We do not see a reduction of Rm62 binding to its sites in *Su(var)3-9* heteroallelic larvae (Figure 6B) suggesting that Rm62 is required for SU(VAR)3-9 binding but not vice versa.

## Discussion

We have identified Rm62 as an interactor with the N-terminus of SU(VAR)3-9. Interestingly, this interaction domain is shared between the SU(VAR)3-9 and eIF2γ [48] and could therefore mediate the interaction between Rm62 and both proteins. We focused our studies on the analysis of the nuclear interaction of Rm62 and SU(VAR)3-9 as it seems to be important for the efficient shut down of highly activated genes such the *hsp70*. In accordance with the previously described role of histone modifications at the heat shock locus [8], we observe a strong H3K9 methylation at the *hsp70* gene before heat shock activation, which disappears after heat shock and slowly reappears when cells recover from heat shock. This methylation is highly dependent on the presence of Rm62 as it is strongly reduced in *Rm62* mutant fly strains. Rm62 mutation not only leads to less H3K9 methylation at the heat shock loci but also leads to a global reduction of the H3K9me2 mark in euchromatin. This suggests a widespread mechanism of methyltransferase recruitment mediated by the interaction between Rm62 and SU(VAR)3-9.

### Histone modifications regulate gene activation and repression at the *hsp70* locus

**Figure 7:**
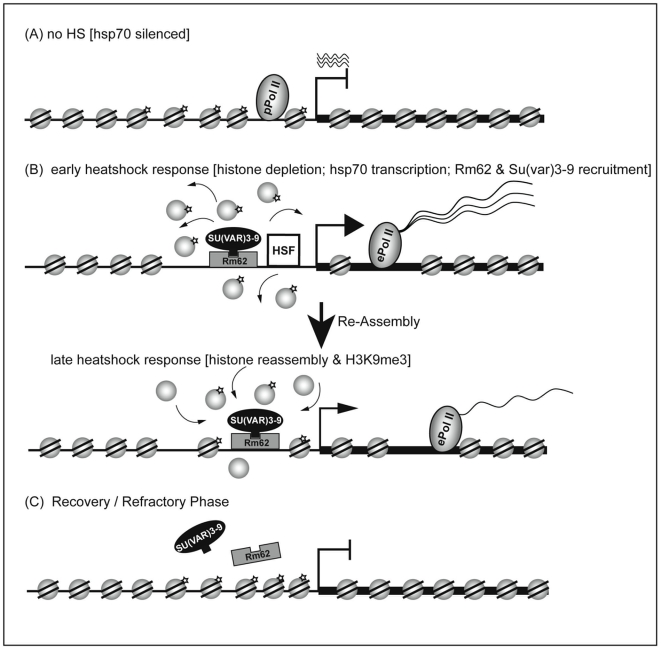
Model for SU(VAR)3-9/Rm62 action at the *hsp70*-locus. (**A**) Under non-heat shock conditions the *hsp70* gene is silenced, but a paused PolII (pPolII) is bound to the promoter and small, kryptic RNAs originate from the transcription start site. (**B**) *Early heat shock response:* upon heat shock, heat shock factor 1 (HSF) is recruited to the *hsp70* promoter, resulting in PolII promoter release and conversion of paused to elongating Pol II (ePolII), leading to the massive generation of *hsp70* RNA. On the chromatin level this state is characterised by histone methylation (H3K4) and subsequent histone depletion. We find that the heat shock also leads to a recruitment of Rm62 and SU(VAR)3-9 to the *hsp70* locus in a RM62 dependent manner. *Late heat shock response:* when the heat shock trigger ceases, reassembling histones get methylated by SU(VAR)3-9 that is already bound. As a consequence of chromatin reassembly, transcription re-initiation and *hsp70-*RNA production are strongly diminished. (**C**) The chromatin state in the *recovery* or *refractory* phase is characterised by a high level of H3K9 methylation and a complete silencing of *hsp70* transcription. SU(VAR)3-9 and Rm62 are released and the gene locus which is prone for another activation cycle.

Histone modifications play a crucial role in regulating gene expression. The *hsp70* locus provides an excellent model promoter for rapidly switching between the on and the off state of transcription and it has been shown to be regulated at multiple levels including histone modification [8], [49]. One of the factors that get recruited to the heat shock promoter immediately after activation is the Rm62, which we identified as an interactor with the histone methyltransferase SU(VAR)3-9. Despite being recruited immediately after heat shock, Rm62 plays a role in transcriptional shut down after removal of the heat shock [39]. It has been suggested that the RNA helicase activity is required for the efficient removal of the RNA from its site of transcription, which in turn is important for the resilencing of the gene [39]. However, a more direct role in the generation of the repressed state could not be excluded. As we observe a strong, Rm62 dependent, recruitment of SU(VAR)3-9 to the promoter after heat shock, which is important for the reestablishment of H3K9 methylated chromatin, we propose that the interaction between the two proteins contributes to the regeneration of a repressive chromatin structure after heat shock. Buszczak and colleagues observe a prolonged phosphorylation of H3S10 at the *hsp70* locus in flies that carry a mutation in Rm62 [39], which may very well be due to a failure of recruiting SU(VAR)3-9 and H3K9 methylation in absence of Rm62. The phosphorylation of H3S10 is severely impaired when the neighboring residue (H3K9) is methylated by SU(VAR)3-9 *in vitro* [26] and *in vivo* [50]. The recruitment of a H3K9 methyltransferase to the *hsp70* gene after heat shock may therefore prevent an efficient phosphorylation of H3S10 thereby favoring the reestablishment of a repressed chromatin structure. At the same time could the increased recruitment of a H3S10 kinase prevent a premature methylation of K9 via the recruited methyltransferases, which may explain the striking kinetic difference we observe between the binding of SU(VAR)3-9 and the accumulation of H3K9 methylated histones (Figure 4 and 5). Our findings may therefore provide another example of a phospho-methyl switch [51] where a strong interdependence of histone methylation and histone phosphorylation on adjacent residues is observed. Alternatively, the lack of histone methylation after heat shock that is seen by immunofluorescence and by ChIP could be due to the fact that histones are completely removed after heat shock and are only reassembled during recovery. In this case the recruitment of SU(VAR)3-9 would lead to an increased local concentration of the methyltransferase at the site of the promoter, which could (re-)methylate the ejected histones leading to the regeneration of a repressed state after heat shock (Figure 7). This may in fact also explain the seemingly paradoxical effect of HP1 localisation at heat shock puffs [45]. The binding data could also suggest that the recruitment of SU(VAR)3-9 is in fact important for gene activation as we find it to bind to the promoter immediately after the induction of transcription. However, we think this is unlikely as we only observe an effect of SU(VAR)3-9 and Rm62 removal on the shut down of *hsp*70 transcription but not on it's induction (Figure 3).

### The role of RNA in *hsp70* regulation

An alternative explanation for the apparent discrepancy between SU(VAR)3-9 binding and H3K9 methylation could be the need for an additional signal for the enzyme to become active. Such a signal could be an external signal such as a posttranslational modification or an internal signal such as the RNA transcribed from the *hsp70* locus itself. Immediately after heat shock a short burst of small RNAs can be detected that are released from the heat shock locus [52]. Considering the fact that Rm62 also plays a role in RNAi mediated silencing [43], this pulse of small RNAs might in fact be the cause for the heat shock dependent recruitment of Rm62 to the *hsp70* locus that we observe. Our data suggest that SU(VAR)3-9 is then recruited to the hsp70 locus via protein-protein interactions where it methylates the histones that are assembled onto the promoter during repression. However, we have not tested the possibility that the RNA stimulates the activity of SU(VAR)3-9, which could also contribute to the delayed histone methylation.

Finally we cannot exclude that, in addition to SU(VAR)3-9, a demethylase is recruited to the *hsp70* locus, which removes the histone methylation from the promoter bound histones. Indeed, the jmjC family member dUTX, which contains a H3K27 specific demthylase associates with the elongating RNA polII enzyme and is recruited to the *hsp70* locus after heat shock [53]. It is very likely that multiple redundant mechanisms play a role in the re-silencing of the *hsp70* genes after heat shock with all the possibilities discussed above being involved. In light of our novel finding of a functional interaction between SU(VAR)3-9 and Rm62 it will be interesting to investigate whether this interaction my also provide a mechanistic link between the shut down of highly active genes and the silencing of repetitive DNA elements via the generation of short non translated transcripts that may help in recruiting a histone methyltransferase. Similar mechanisms have been shown to operate in *S. pombe* [54], [55] but were so far not identified in higher eukaryotes.

## Supporting Information

Figure S1 **Knockdown of Su(var)3-9 and Rm62 does not effect cell division.** Drosophila SL2 cells were treated with specific dsRNA against Su(var)3-9 (□), Rm62 (▴) or a combination of both (⊥). dsRNA against Glutathion-S-Transferase (GST; □) served as an internal control. At the day of RNAi treatment (day 0), cells (1,25×10^6^) were seeded in Schneider's Drosophila medium supplemented with 10% fetal calf serum and incubated at 26°C. Cell numbers were checked three and six days after RNAi treatment.
(PDF)
