# Supplementary figures and images for: The RNA Helicase Rm62 Cooperates with SU(VAR)3-9 to Re-Silence Active Transcription in Drosophila melanogaster
Source: PLoS One. 2011 Jun 2;6(6):e20761. doi: 10.1371/journal.pone.0020761 (PMC3107242; doi:10.1371/journal.pone.0020761)

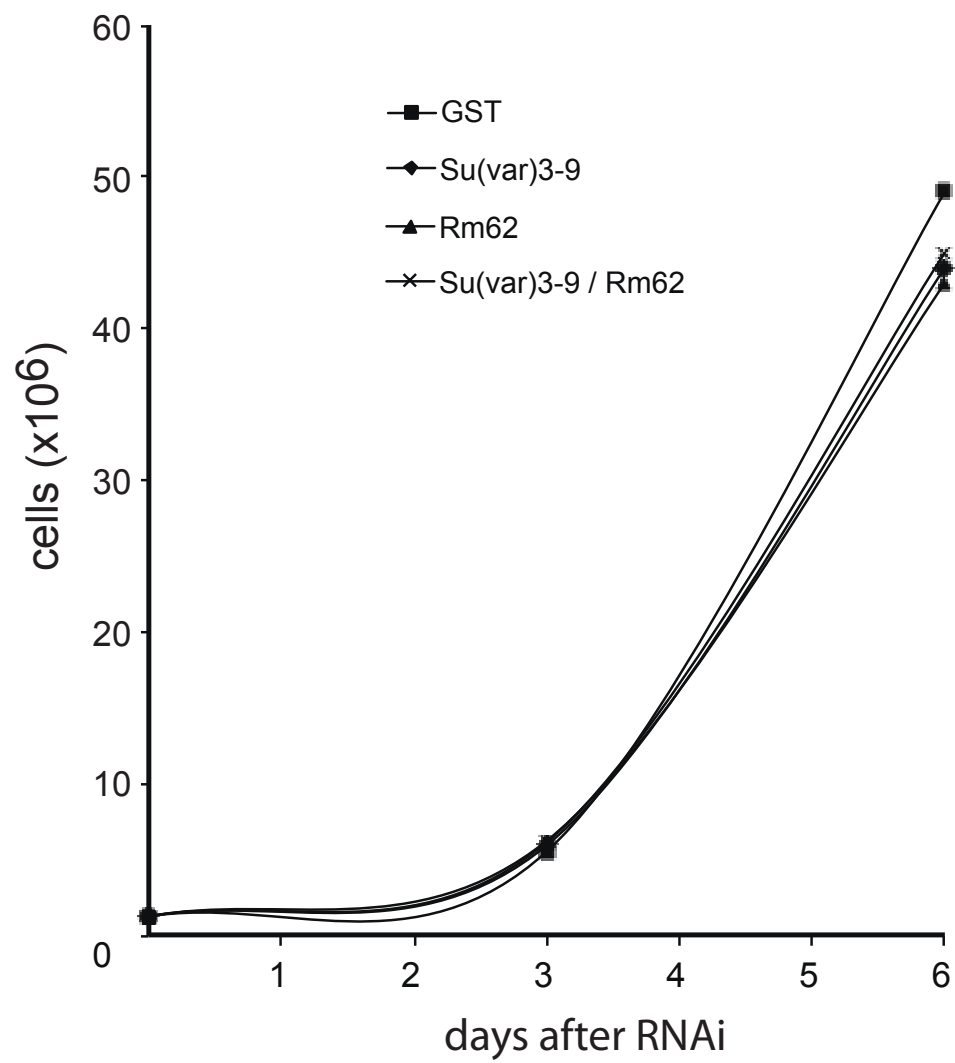

Figure S1 Boeke et al

Supplement: Figure S1 — Knockdown of Su(var)3-9 and Rm62 does not effect cell division. Drosophila SL2 cells were treated with specific dsRNA against Su(var)3-9 (□), Rm62 (▴) or a combination of both (⊥). dsRNA against Glutathion-S-Transferase (GST; □) served as an internal control. At the day of RNAi treatment (day 0), cells (1,25×10^6^) were seeded in Schneider's Drosophila medium supplemented with 10% fetal calf serum and incubated at 26°C. Cell numbers were checked three and six days after RNAi treatment. (PDF) [file pone.0020761.s001.pdf]
